# Supplementary material for: DNA polymerase ε harmonizes topological states and R-loops formation to maintain genome integrity in Arabidopsis
Source: Nat Commun. 2023 Nov 27;14:7763. doi: 10.1038/s41467-023-43680-7 (PMC10682485; doi:10.1038/s41467-023-43680-7)
Supplement: Supplementary file 1 — Supplementary Information [file 41467_2023_43680_MOESM1_ESM.pdf]

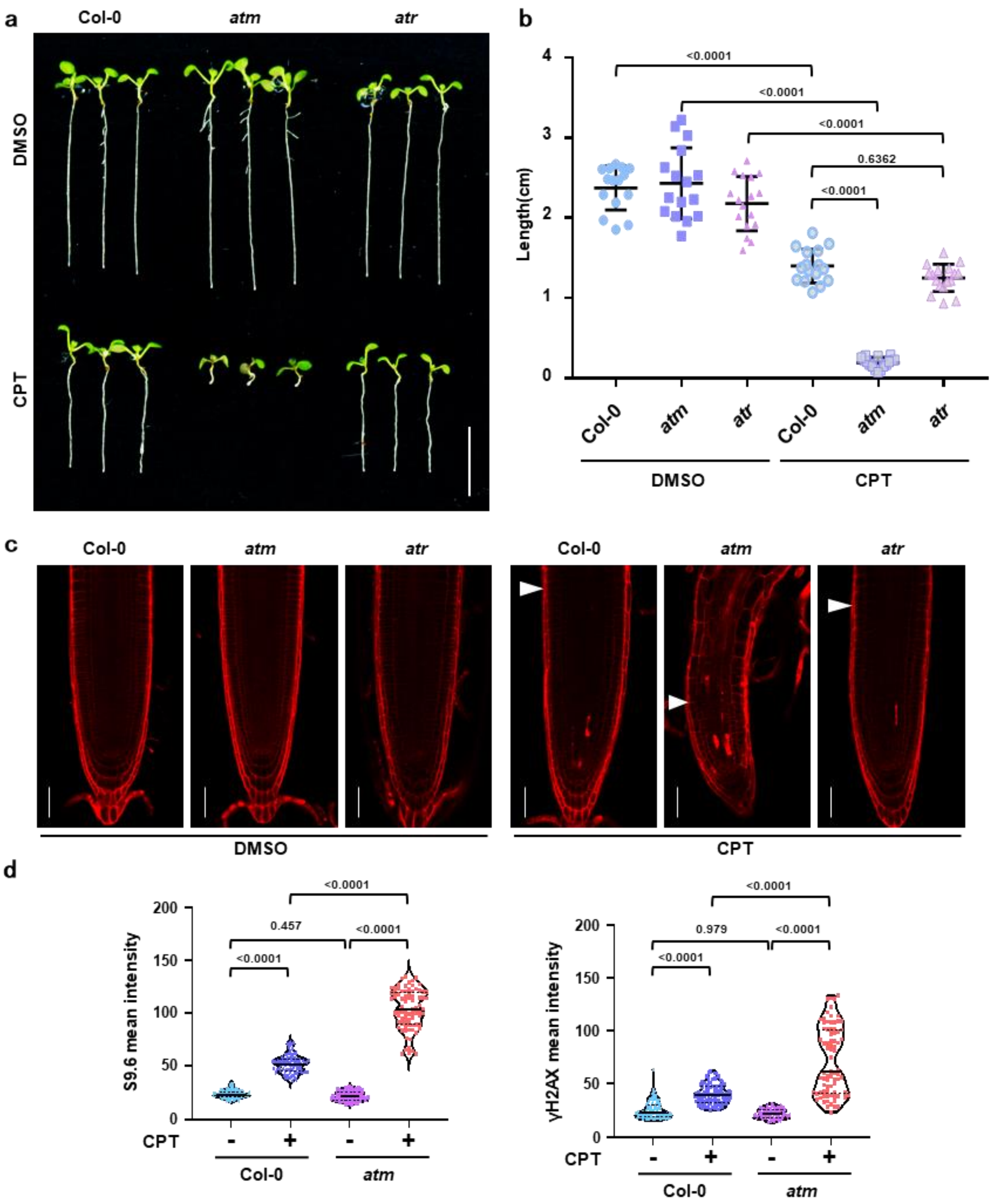

Supplementary Figure 1. TOP1i inhibits root development in *atm* but not in *atr*

(a) Phenotypes of plant roots treated with DMSO (control) or 30 nM CPT (TOP1i). for 7 days. Scale bar: 1 cm. **(b)** The quantitative data of root lengths for Supplementary Fig.1a. Lines represent mean ± SD, n=15 (one-way ANOVA). Source data are provided as a Source Data file. **(c)** PI staining corresponding to Supplementary Fig.1a shows that TOP1i resulted in root meristem cell death. The white triangle indicates the boundary of the meristematic zone and elongation zone. Scale bar: 50 μm. **(d)** R-loop levels indicated by S9.6 and DNA damage levels indicated by γH2AX in mutants related to Fig. 1e. Mean intensity was evaluated using Image J software. Lines represent mean ± SD, n=80 (one-way ANOVA). Source data are provided as a Source Data file.

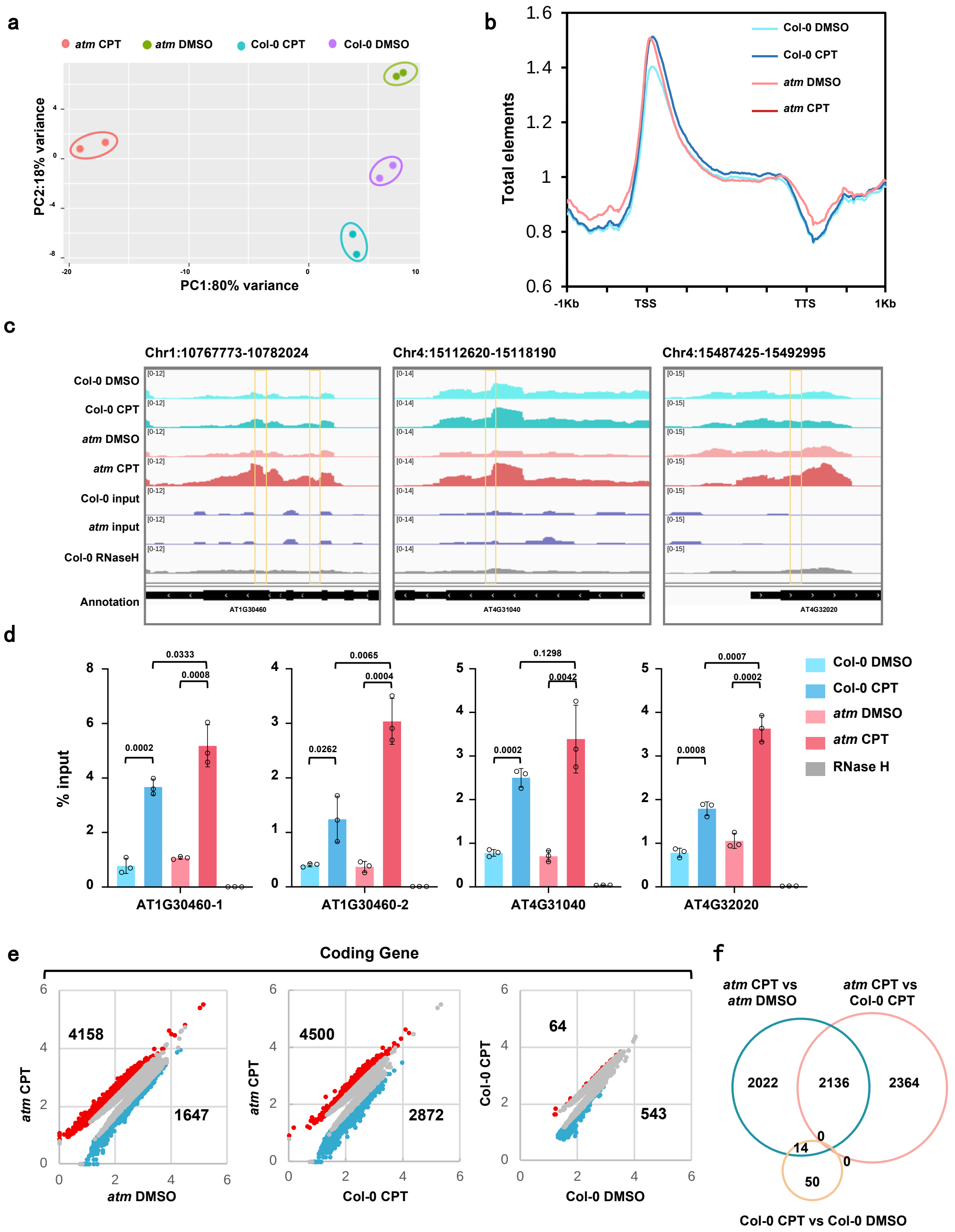

Supplementary Figure 2. Genomic R-loops change after TOP1i treatment, especially in *atm*

(a) Principal Components Analysis of ssDRIP-seq signals in root tips of Col-0 and *atm* treated with DMSO or 1 μM CPT for 2 hours. Each dot represents one sample, and the same color represents two biological repeats. **(b)** Metaplots of unstranded R-loops centered on total annotated genes. TSS: transcription start site, TTS: transcription termination site. R-loop levels were plotted with the average score of two biological replicates. **(c)** Snapshots of ssDRIP-seq in Col-0 and *atm* with or without CPT treatment. Col-0 and *atm* input samples were used as the

control, and Col-0 with RNase H treatment was used as the negative control. Yellow boxes indicate the tested regions by DRIP-qPCR in Supplementary Fig.2d. **(d)** DRIP-qPCR shows R-loop levels in Col-0 and *atm* with and without CPT treatment. Bars in the plot represent mean ± SD with three replicates. (one-way ANOVA). Source data are provided as a Source Data file. **(e)** Scatterplots showing the significantly up-regulated and down-regulated R-loop gene numbers. Normalized read counts are shown as $\log_{10}(n + 1)$. Red dots: q-value < 0.05, $\log_2FC > 1$; blue dots: q-value < 0.05, $\log_2FC < -1$; gray dots: other. Source data are provided as a Source Data file. **(f)** Venn diagram of significantly up-regulated R-loop genes in the samples shown in Supplementary Fig.2e.

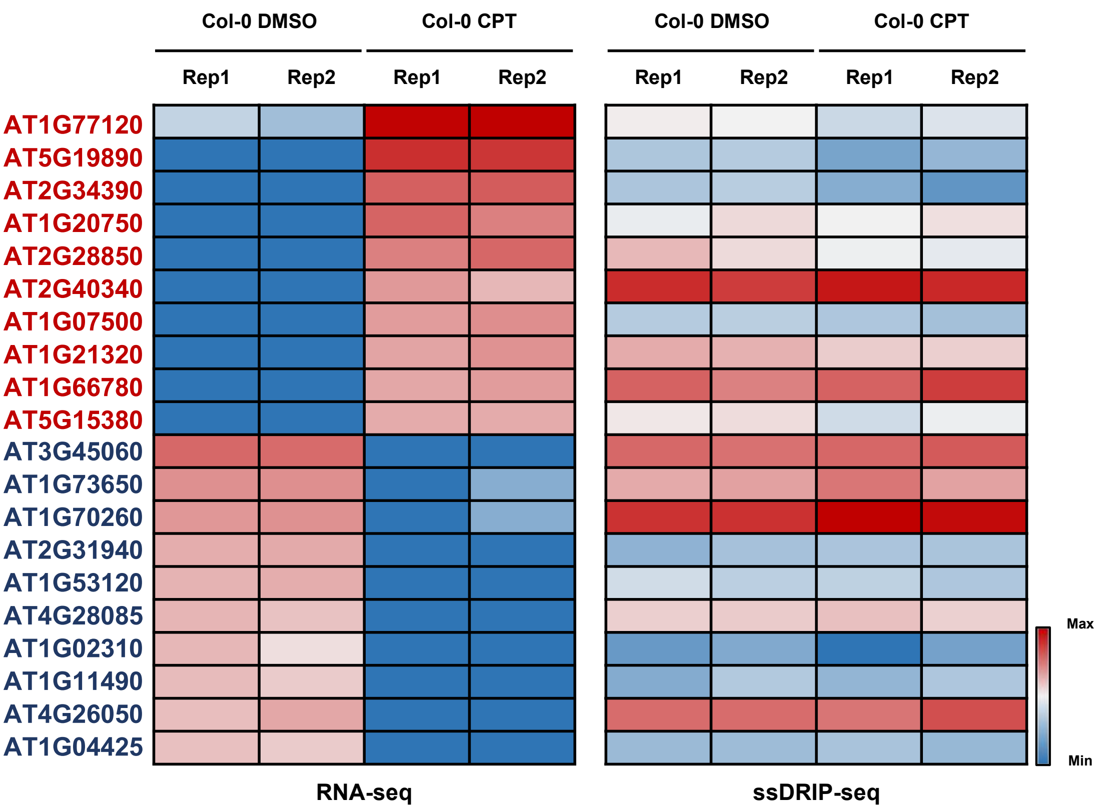

Supplementary Figure 3. TOP1i-induced R-loops are not associated with transcription

Heatmaps of RNA-seq and ssDRIP-seq signals in the top 10 up-regulated (marked with red) and down-regulated genes (marked with blue) in Col-0 after CPT treatment. Two biological replicates are shown.

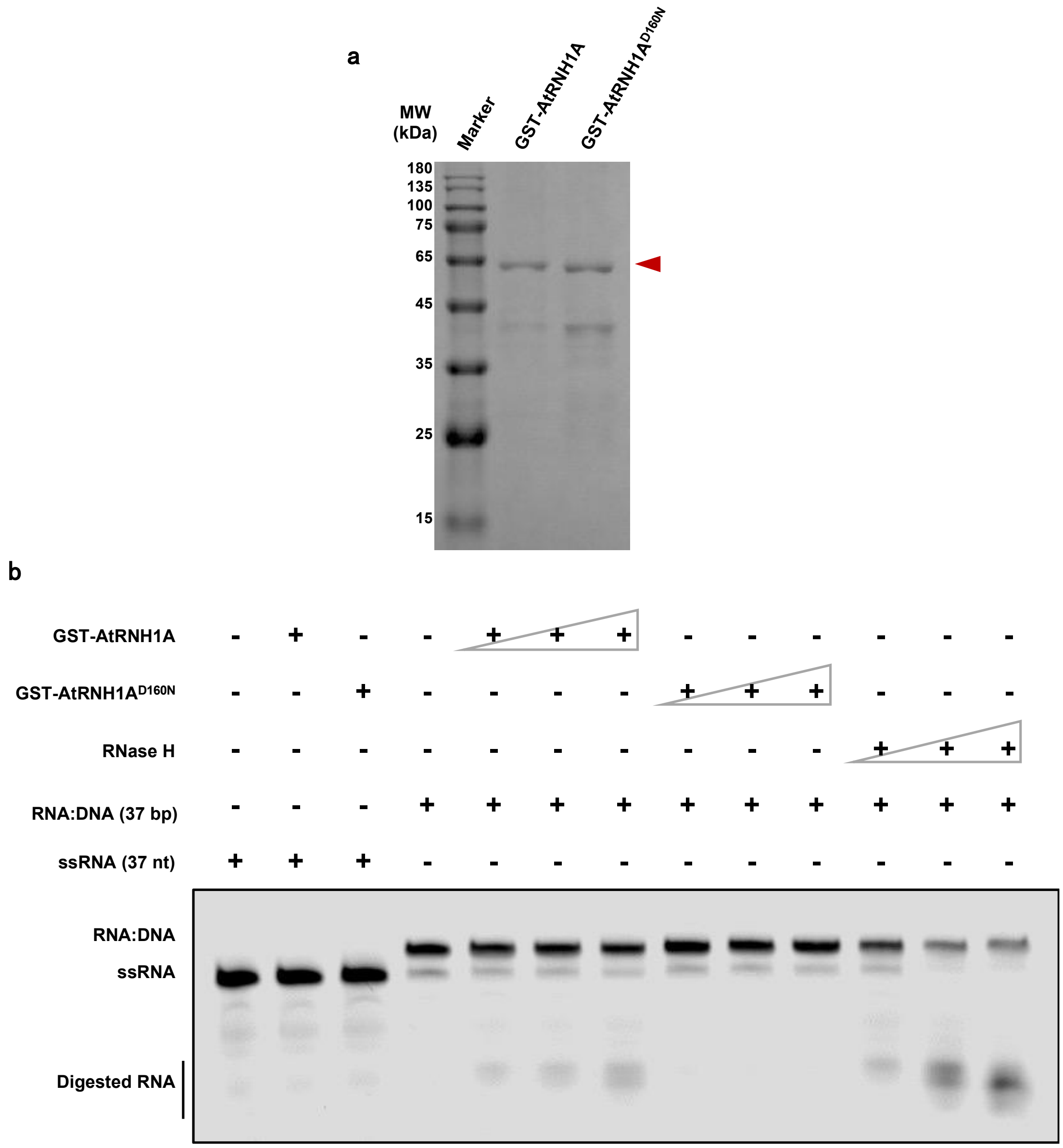

Supplementary Figure 4. AtRNH1A^{D160N} blocks RNase H activity

(a) The SDS-PAGE gel staining showing the purified GST-AtRNH1A and GST-AtRNH1A^{D160N} proteins. The red triangle indicates the proteins (~ 60 kDa). **(b)** Analysis of AtRNH1A in vitro RNase H activity by electrophoretic mobility shift assay with 1 μM synthetic RNA:DNA hybrids. The triangles from left to right indicated the increased concentrations of GST-AtRNH1A, GST-AtRNH1A^{D160N} at 1, 2.5 and 5 pM, and commercial RNase H at 1, 2.5 and 5 U, respectively.

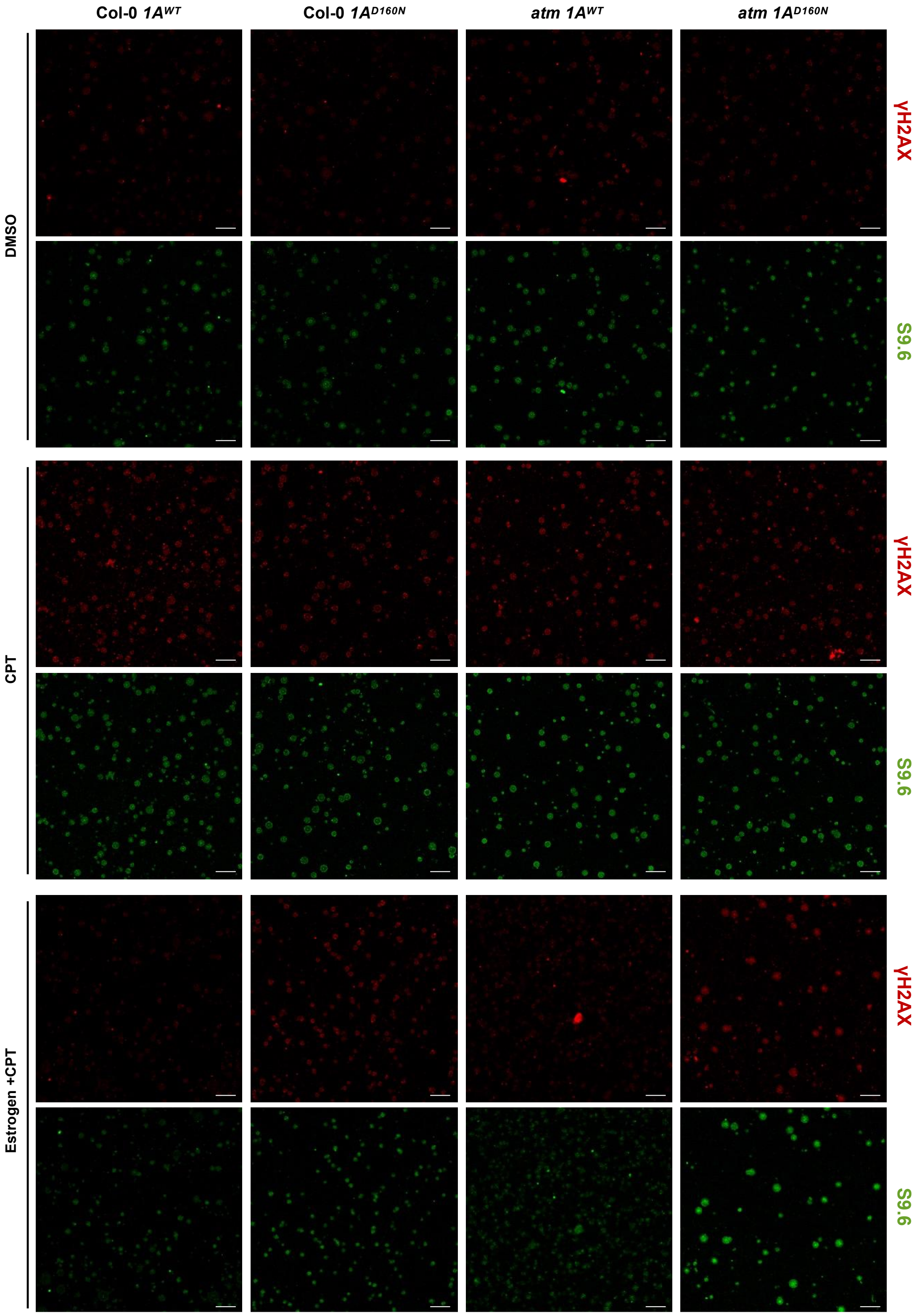

Supplementary Figure 5. *AtRNH1A* overexpression decreased TOP1i-induced R-loops and γH2AX

Immunolabeling of root tip nuclei from materials corresponding to Fig.2a using R-loop antibody S9.6 and DSB marker γH2AX. Scale bar: 20 μm. Experiments were repeated three times with similar results.

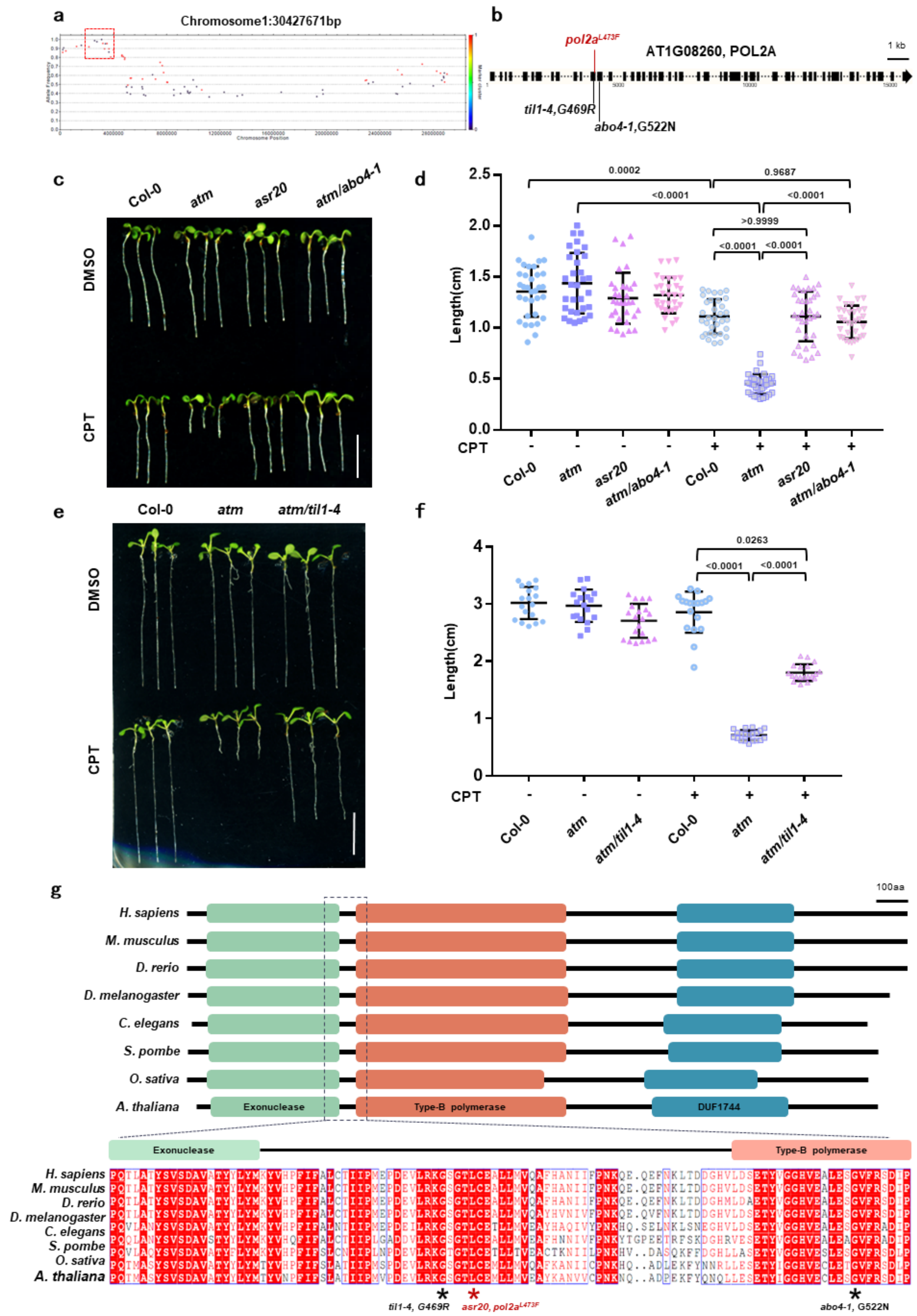

Supplementary Figure 6. ***ASR20*** **encodes DNA polymerase ε catalytic subunit POL2A**

(a) Genome localization analysis of *asr20* mutation sites using SHOREmap. The candidate mutation region (red dotted box) is on the short arm of chromosome 1. **(b)** Gene model of POL2A showing mutation sites. The *asr20* screened in this study is a C to T mutation at position 3939 changing Leu (L) 473 to Phe (F); two closing alleles are a G to A mutation at position 3927

changing Gly (G) 469 to Arg (R), and a G to A mutation at position 4171 changing Gly (G) 522 to Asp (N). **(c)** Root phenotypes of 5-day-old seedlings treated with DMSO and 30 nM CPT. Scale bar: 1 cm. **(d)** The quantitative data of root lengths for Supplementary Fig.6c. Lines represent mean ± SD, n=25 (one-way ANOVA). Source data are provided as a Source Data file. **(e)** Root phenotypes of 7-day-old seedlings treated with DMSO and 30 nM CPT. Scale bar: 1 cm. **(f)** The quantitative data of root lengths for Supplementary Fig.6e. Lines represent mean ± SD, n=15 (one-way ANOVA). Source data are provided as a Source Data file. **(g)** Conservation analysis of POL2A functional domains. Domains are predicted according to the conserved domain database from NCBI. The 3′ to 5′ exonuclease domain, DNA type-B polymerase catalytic domain, and DUF1714 domain (domain of the unknown function) are shown with different colors. The amino acid residues of the junction region between the exonuclease domain and polymerase domain were highly conserved. Mutation sites of three weak alleles are indicated by stars.

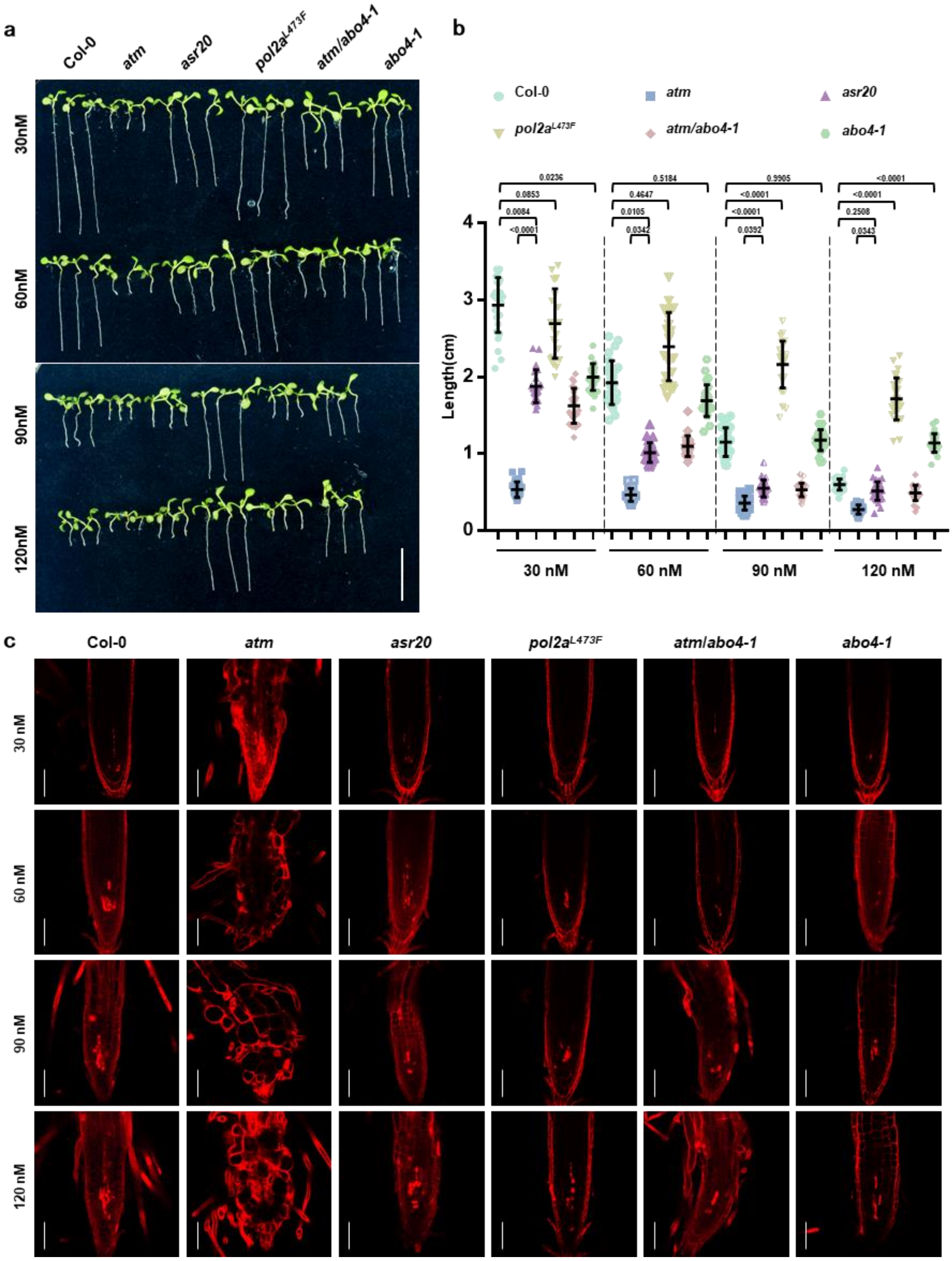

Supplementary Figure 7. ***pol2a*** **mutants are hyper-resistant to high concentrations of CPT**

(a) Root phenotypes of 7-day-old Col-0, *atm*, *asr20*, *pol2a*^{L473F}, *atm/abo4-1*, and *abo4-1* after treatment with increasing CPT concentrations (30, 60, 90, and 120 nM). Scale bar: 1 cm. **(b)** The quantitative data of root lengths for Supplementary Fig.7a. Lines represent mean ± SD, n=20 (one-way ANOVA). Source data are provided as a Source Data file. **(c)** PI staining corresponding to Supplementary Fig.7a shows root morphology of seedlings under increasing CPT concentrations (30, 60, 90,120 nM, respectively). Scale bar:100 μm.

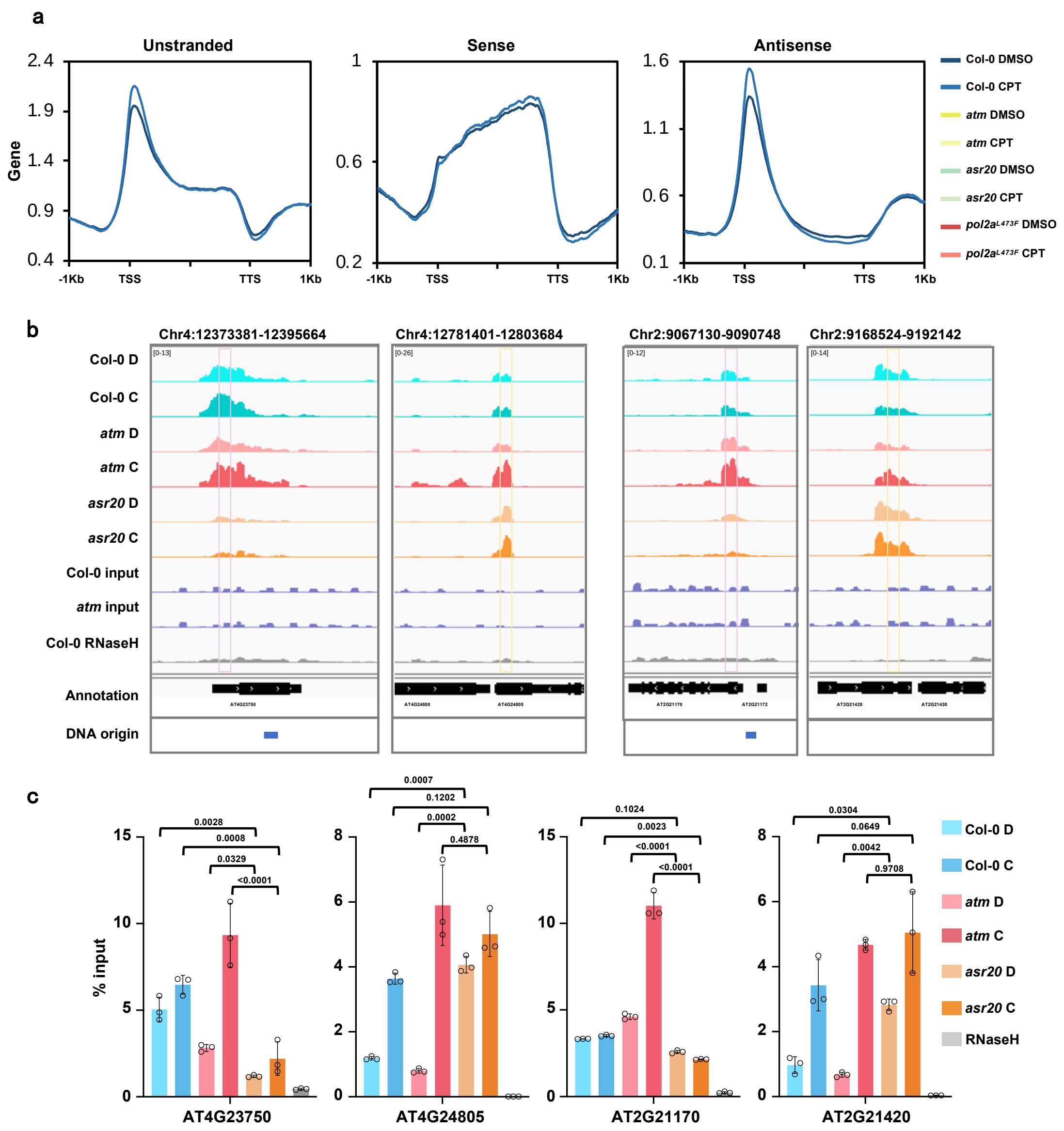

Supplementary Figure 8. R-loops are increased near DNA origins upon TOP1i in *atm*

(a) Metaplot results of unstranded, sense, and antisense R-loops centered on protein coding genes in Col-0 and mutants with DMSO or CPT treatment. TSS: transcription start site, TTS: transcription termination site. R-loop levels were plotted with the average score of two biological replicates. **(b)** Snapshots of R-loop levels in regions near and away from DNA origins in Col-0, *atm*, and *asr20*. DNA origin sites are indicated with blue lines at the bottom. Plants treated with DMSO (d) or CPT (c) are indicated on the left. Col-0 and *atm* input samples were used as the control, and Col-0 with RNase H treatment was used as the negative control. **(c)** DRIP-qPCR of root tips treated with DMSO or 1 μM CPT for 2 hours from Col-0, *atm*, and *asr20* at three DNA origins shown in Supplementary Fig.8a. Data are normalized to input, results were shown as mean values ± SD; circles show the original data from three replicates (One-way ANOVA). RNase H treatment was used as the negative control. Source data are provided as a Source Data file.

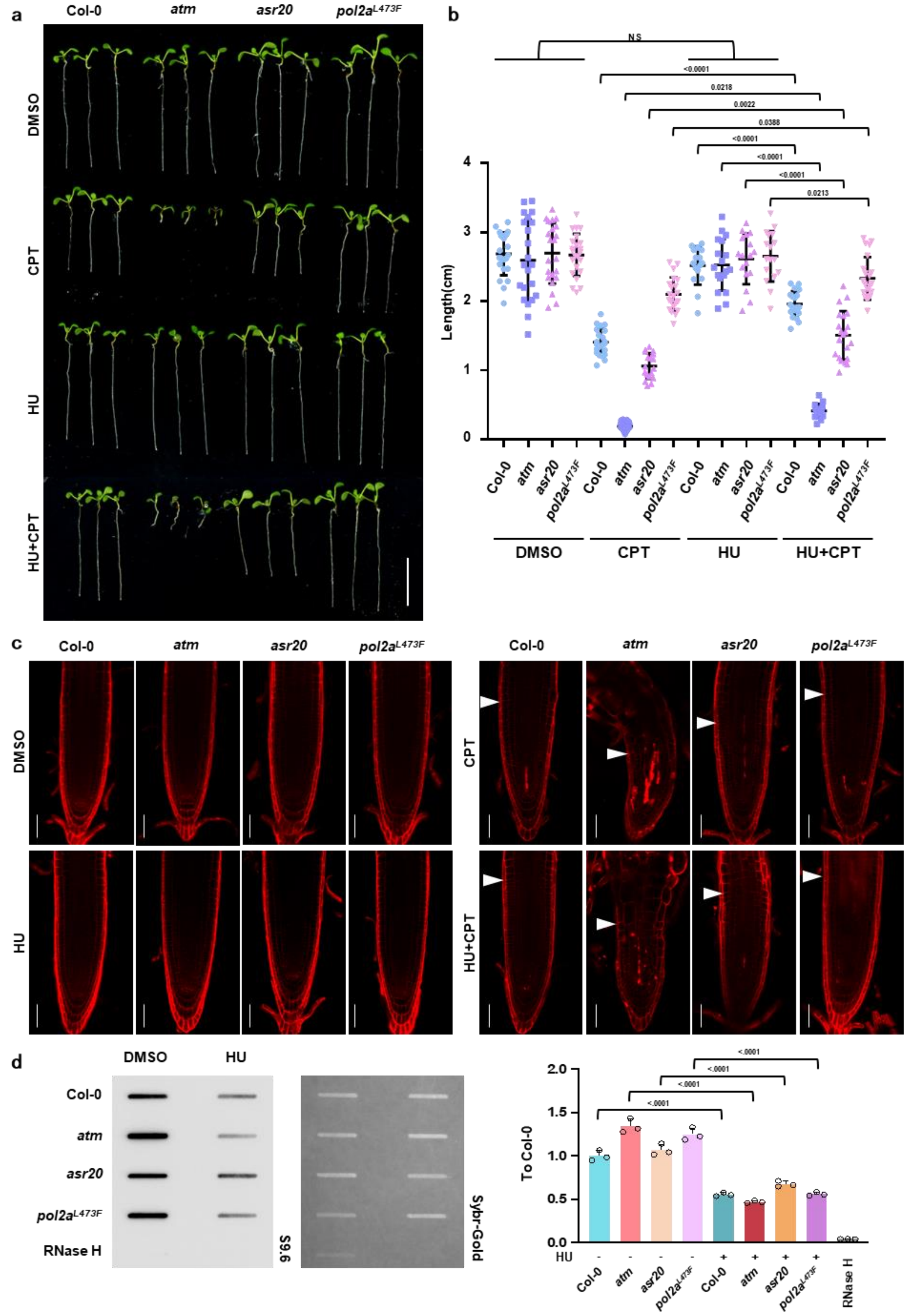

Supplementary Figure 9. DNA replication stress modulates topoR-loops

(a) Root length phenotypes after treatment with 0.25 mM replication inhibitor hydroxyurea (HU). Scale bar: 1 cm. **(b)** The quantitative data of root lengths for Supplementary Fig.9a. Lines represent mean ± SD, n=20, NS: no significant difference (one-way ANOVA; Individual p-values

are detailed in Source Data file). Source data are provided as a Source Data file. **(c)** PI staining corresponding to Supplementary Fig.9a shows that 0.25 mM HU had no inhibiting effects on root development but significantly reduced TOP1i-induced root stem cell death and improved root morphology in *atm.* White triangles indicate the boundary of the meristematic zone and elongation zone. Scale bar: 50 μm. **(d)** Genomic DNA of root tips (with and without 2 hours of 2.5 mM HU treatment) was detected by slot-blot assay using the antibody S9.6. The SYBR-gold staining was used as the loading control. Band signals of each sample are calculated three times with Image J. Bars in the plot represent mean ± SD (one-way ANOVA). Source data are provided as a Source Data file.

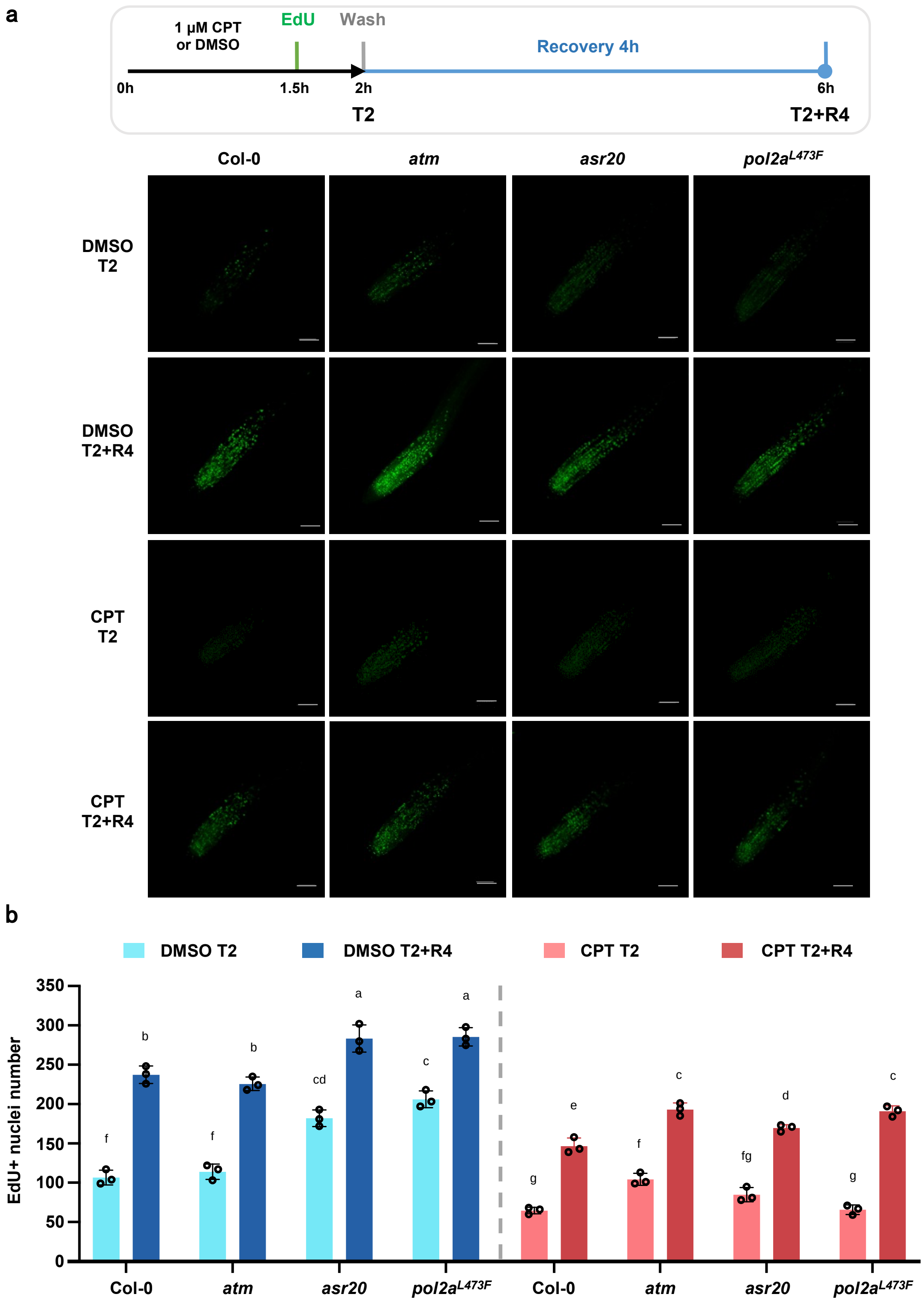

Supplementary Figure 10. POL2A mutation disturbs DNA replication

(a) Brief schematic workflow for EdU-labeling to detect DNA replication of root tips in Col-0 and mutants. 5-day-old seedlings were treated with DMSO or 1 μM CPT for 1.5 h, then labelled with 10 μM EdU for 30 min. Half of root tips were collected for EdU labeling. The remainder were transferred to 1/2 MS liquid medium containing 10 μM EdU to recover growth for 4 h, and then collected for EdU labeling. **(b)** The quantitative data of nuclei labeled by EdU related to

Supplementary Fig.10a. 3 roots were counted in each sample. Lines represent mean ± SD, Different letters indicate significant differences (one-way ANOVA; Individual p-values are detailed in Source Data file). Source data are provided as a Source Data file.

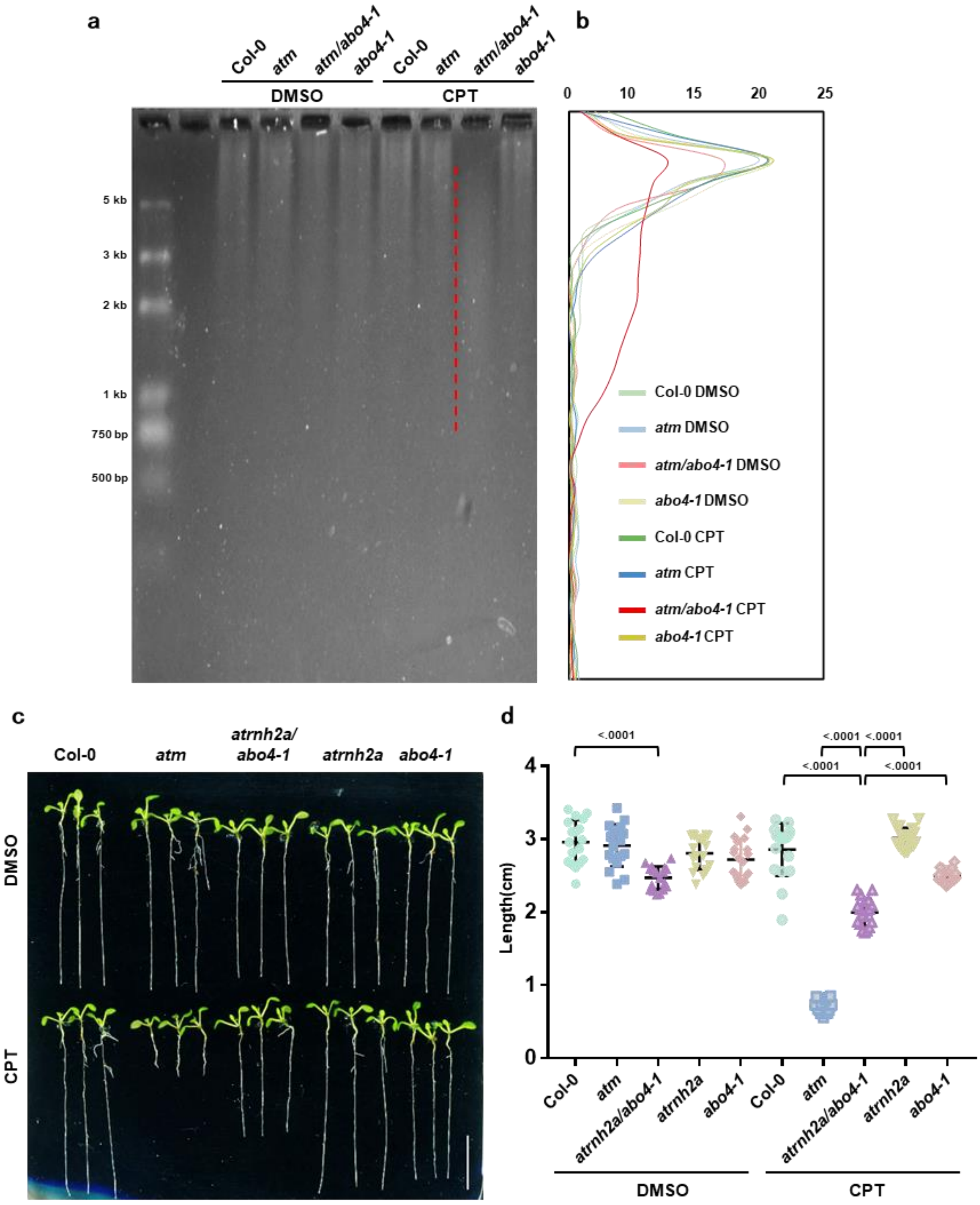

Supplementary Figure 11. POL2A and ATM guard the fidelity of DNA replication

(a) Ribonucleotide incorporation detection with RNase HII cleavage products of genomic DNA extracted from Col-0, *atm*, *atm/abo4-1,* and *abo4-1* treated with DMSO or 30 nM CPT for 7 days, the rNTPs incorporation ratio was elevated in the *atm/abo4-1* genome after CPT treatment as 2-5 kb DNA fragments (dashed red line) were predominantly detected. **(b)** Densitometry plot of lanes corresponding to Supplementary Fig.11a. **(c)** Root phenotypes of 8-day-old Col-0 and mutants treated with DMSO or 30 nM CPT. Scale bar: 1 cm. **(d)** The quantitative data of root lengths for Supplementary Fig.11c. Lines represent mean ± SD, n=20 (one-way ANOVA). Source data are provided as a Source Data file.

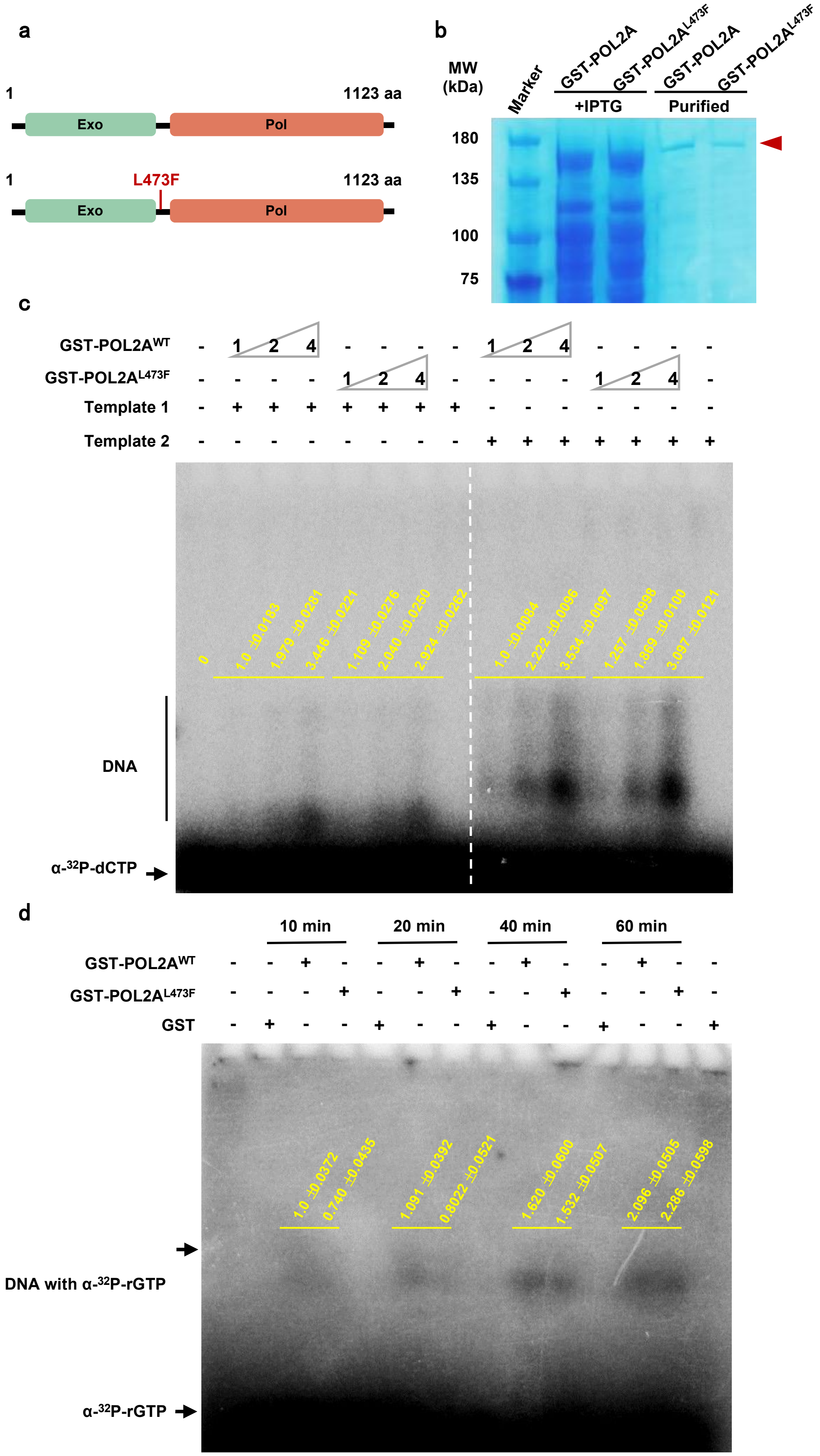
a
1
1123 aa
Exo
Pol
1
L473F
1123 aa
Exo
Pol
b
MW (kDa)
Marker
GST-POL2A
GST-POL2A^L473F
GST-POL2A
GST-POL2A^L473F
+IPTG
Purified
180
135
100
75
c
GST-POL2A^WT
GST-POL2A^L473F
Template 1
Template 2
1 2 4
0
1.0 ±0.0183
1.979 ±0.0281
3.446 ±0.0221
1.109 ±0.0276
2.040 ±0.0250
2.924 ±0.0262
1.0 ±0.0084
2.222 ±0.0096
3.534 ±0.0097
1.257 ±0.0998
1.869 ±0.0100
3.097 ±0.0121
DNA
α-32P-dCTP
d
10 min
20 min
40 min
60 min
GST-POL2A^WT
GST-POL2A^L473F
GST
1.0 ±0.0372
0.740 ±0.0435
1.091 ±0.0392
0.8022 ±0.0521
1.620 ±0.0600
1.532 ±0.0507
2.096 ±0.0505
2.286 ±0.0598
DNA with α-32P-rGTP
α-32P-rGTP

Supplementary Figure 12. Impact of POL2A mutation on polymerase activity.

(a) Diagrams showing the wild type and point mutation form of recombinant truncated POL2A (1-1123 aa). Exo: exonuclease domain, Pol: polymerase catalytic domain. **(b)** The SDS-PAGE detected gel staining showing the recombinant GST-POL2A and GST-POLAL473F proteins before and after purification. The red triangle indicates the proteins (~160 kDa). **(c)** DNA synthesis by GST-POL2A^{WT} and GST-POL2A^{L473F} using two DNA templates. The amounts of proteins with 1, 2 and 4 pmol in assays as indicated. The synthesized DNA was labeled with [α-32P]-dCTP. The sequences of DNA templates are listed in Supplementary Data 1. The band signals of each sample are calculated three times with Image J. Values above the band represent mean ± SD. **(d)** rNTPs incorporation detection during DNA synthesis by GST-POL2A^{WT} and GST-POL2A^{L473F}. The GST protein was used as the negative control. Proteins were present at 1 pmol. The rNTPs-incorporated DNA was labeled with [α-32P]-rGTP. The band signals of each sample are calculated three times with Image J. Values above the band represent mean ± SD.

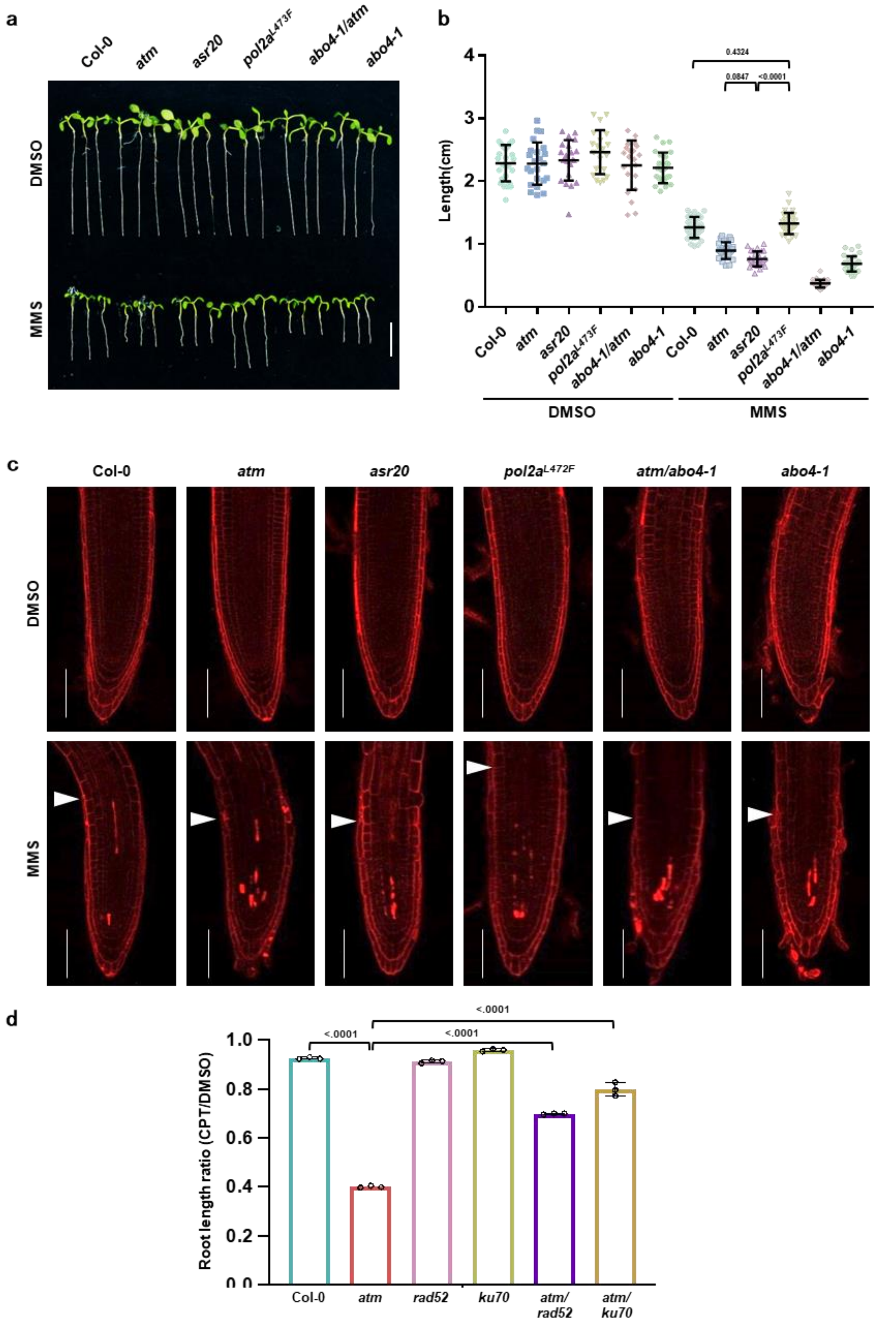

Supplementary Figure 13. ***pol2a^{L473F}*** **shows resistance to MMS**

(a) Root length phenotypes in mutants treated with 100 ppm methyl methanesulfonate (MMS). Scale bar: 1 cm. **(b)** The quantitative data of root lengths for Supplementary Fig.13a. Lines represent mean ± SD (one-way ANOVA). Source data are provided as a Source Data file. **(c)**

PI staining shows dead cells and root structures corresponding to Supplementary Fig.13a. The white triangles indicate the boundary of the meristematic zone and elongation zone. Scale bar: 100 µm. **(d)** Root length ratio of different samples after CPT treatment. The root length in each sample shown in Figure 8F was used for calculating the ratio. The 15 roots of each sample were randomly divided into 3 parts, and the average value of each part was used for statistics. Lines represent mean ± SD, n=3 (one-way ANOVA). Source data are provided as a Source Data file.
