# Supplementary material for: DNA polymerase ε harmonizes topological states and R-loops formation to maintain genome integrity in Arabidopsis
Source: Nat Commun. 2023 Nov 27;14:7763. doi: 10.1038/s41467-023-43680-7 (PMC10682485; doi:10.1038/s41467-023-43680-7)
Supplement: Supplementary file 3 — Description of Additional Supplementary Files [file 41467_2023_43680_MOESM3_ESM.pdf]

Description of Additional Supplementary Files

File Name: Supplementary Data 1
Description: Oligos used for genotyping, DRIP-qPCR, EMSA and DNA synthesis.
