# Supplementary material for: DNA polymerase ε harmonizes topological states and R-loops formation to maintain genome integrity in Arabidopsis
Source: Nat Commun. 2023 Nov 27;14:7763. doi: 10.1038/s41467-023-43680-7 (PMC10682485; doi:10.1038/s41467-023-43680-7)
Supplement: Supplementary file 5 — Reporting Summary [file 41467_2023_43680_MOESM5_ESM.pdf]

nature portfolio

Corresponding author(s): Qianwen Sun

Last updated by author(s): Oct 31, 2023

Reporting Summary

Nature Portfolio wishes to improve the reproducibility of the work that we publish. This form provides structure for consistency and transparency in reporting. For further information on Nature Portfolio policies, see our Editorial Policies and the Editorial Policy Checklist.

Statistics

For all statistical analyses, confirm that the following items are present in the figure legend, table legend, main text, or Methods section.

n/a	Confirmed	
☐	☒	The exact sample size (*n*) for each experimental group/condition, given as a discrete number and unit of measurement
☐	☒	A statement on whether measurements were taken from distinct samples or whether the same sample was measured repeatedly
☐	☒	The statistical test(s) used AND whether they are one- or two-sided<br>*Only common tests should be described solely by name; describe more complex techniques in the Methods section.*
☒	☐	A description of all covariates tested
☐	☒	A description of any assumptions or corrections, such as tests of normality and adjustment for multiple comparisons
☐	☒	A full description of the statistical parameters including central tendency (e.g. means) or other basic estimates (e.g. regression coefficient) AND variation (e.g. standard deviation) or associated estimates of uncertainty (e.g. confidence intervals)
☐	☒	For null hypothesis testing, the test statistic (e.g. *F*, *t*, *r*) with confidence intervals, effect sizes, degrees of freedom and *P* value noted<br>*Give P values as exact values whenever suitable.*
☒	☐	For Bayesian analysis, information on the choice of priors and Markov chain Monte Carlo settings
☒	☐	For hierarchical and complex designs, identification of the appropriate level for tests and full reporting of outcomes
☒	☐	Estimates of effect sizes (e.g. Cohen's *d*, Pearson's *r*), indicating how they were calculated

Our web collection on statistics for biologists contains articles on many of the points above.

Software and code

Policy information about availability of computer code

Data collection	Zeiss LSM780/880 microscope for staining<br>Typhoon FLA 9500 scanner for EMSA results detection<br>UV trans-illuminator for DNA gel visualization<br>GE ImageQuant LAS 4000 for western-blot imaging<br>Roche LightCycler480 for qPCR<br>Illumina HiSeq 2500 for NGS sequencing
Data analysis	Image J 1.52 for root length and fluorescence intensity measurement<br>GraphPad Prism 9 for statistical data analysis<br>ZEN 2.3 image software for staining image analysis<br>FastQC v0.11.9 for quality control of ssDRIP-seq data<br>Trim_galore v0.6.5 for adapter cutting and tail trimming of ssDRIP-seq data<br>Bowtie2 2.3.5.1 for ssDRIP-seq data alignment<br>bedtools v2.29.2 for genome annotation of ssDRIP peaks<br>Samtools 1.7 for strand splitting of ssDRIP-seq processing<br>Picard 2.24.2 for duplicates removing of ssDRIP reads<br>MACS2 2.2.7.1 for peak calling of ssDRIP data<br>DESeq2 1.32.0 for differentially expressed gene analysis of ssDRIP<br>deepTools 3.5.0 for nonquantified normalization and plotprofiling

For manuscripts utilizing custom algorithms or software that are central to the research but not yet described in published literature, software must be made available to editors and reviewers. We strongly encourage code deposition in a community repository (e.g. GitHub). See the Nature Portfolio guidelines for submitting code & software for further information.

Data

Policy information about availability of data

All manuscripts must include a data availability statement. This statement should provide the following information, where applicable:

- Accession codes, unique identifiers, or web links for publicly available datasets
- A description of any restrictions on data availability
- For clinical datasets or third party data, please ensure that the statement adheres to our policy

The sequencing data and processed files in this study have been deposited in the NCBI Gene Expression Omnibus (GEO) database [https://www.ncbi.nlm.nih.gov/gds] under accession number GSE216170 [https://www.ncbi.nlm.nih.gov/geo/query/acc.cgi?acc=GSE216170]. The other datasets including the histone marker region and DNA replication region used in this study are from NCBI GEO database GSE28398 [https://www.ncbi.nlm.nih.gov/geo/query/acc.cgi?acc=GSE28398] and GSE21928 [https://www.ncbi.nlm.nih.gov/geo/query/acc.cgi?acc=GSE21928]. Source data are provided with this paper.

Human research participants

Policy information about studies involving human research participants and Sex and Gender in Research.

Reporting on sex and gender	not applicable
Population characteristics	not applicable
Recruitment	not applicable
Ethics oversight	not applicable

Note that full information on the approval of the study protocol must also be provided in the manuscript.

Field-specific reporting

Please select the one below that is the best fit for your research. If you are not sure, read the appropriate sections before making your selection.

☒ Life sciences ☐ Behavioural & social sciences ☐ Ecological, evolutionary & environmental sciences

For a reference copy of the document with all sections, see nature.com/documents/nr-reporting-summary-flat.pdf

Life sciences study design

All studies must disclose on these points even when the disclosure is negative.

Sample size	Sample sizes used for each experiment are indicated in the corresponding figures and legends.
Data exclusions	No data were excluded.
Replication	Two or three biologically independent replicates were used for the analysis as indicated in the figure legends, and the replicates were all successful and repeatable.
Randomization	Enrichment analysis of ssDRIP peaks with other genomic features was performed based on randomized permutation tests, values were determined by observed-values / mean of permutation-values from 1,000 replications.<br>For each experiment, the Arabidopsis seedlings were grown in the same conditions and randomly allocated into the indicated experimental groups.
Blinding	Blinding was not applied during the experiments since the same experimental procedures and analysis pipelines were performed in parallel for samples with different genetic backgrounds in each experimental group, which is not suspected to detection bias.

Reporting for specific materials, systems and methods

We require information from authors about some types of materials, experimental systems and methods used in many studies. Here, indicate whether each material, system or method listed is relevant to your study. If you are not sure if a list item applies to your research, read the appropriate section before selecting a response.

Materials & experimental systems

n/a	Involved in the study
☐	☒ Antibodies
☒	☐ Eukaryotic cell lines
☒	☐ Palaeontology and archaeology
☒	☐ Animals and other organisms
☒	☐ Clinical data
☒	☐ Dual use research of concern

Methods

n/a	Involved in the study
☐	☒ ChIP-seq
☒	☐ Flow cytometry
☒	☐ MRI-based neuroimaging

Antibodies

Antibodies used

S9.6 monoclonal antibody: 8μg for each sample in ssDRIP, 1:1000 dilution for Slot blot, 1:200 for immunostaining.
Anti-γH2A.X-S139 (AP1267, ABclonal): 1:3000 dilution for western blot, 1:200 dilution for immunostaining.
Anti-FLAG antibody (F1804, Sigma-Aldrich): 1:5000 dilution for western blot.
Goat anti-rabbit-HRP (BE0101, Easybio): 1:5000 dilution for western blot.
Goat anti-mouse-HRP (BE0102, Easybio): 1:5000 dilution for western blot.
Anti-BrdU antibody (B35128, Invitrogen): 1:200 dilution for immunostaining.
Anti-Rabbit-Alexa Fluor 555 (ab150078, Abcam): 1:200 dilution for immunostaining.
Goat-anti-mouse-DyLight 488 (ab96871, Abcam): 1:200 dilution for immunostaining.

Validation

The S9.6 antibody was purified from S9.6 hybridoma cell supernatant by QinLi, which is validated in publications (PMID:31914390; 35550870).
Validation statements of other commercial antibodies are provided by the companies.
Anti-γH2A.X-S139 https://abclonal.com.cn/catalog/AP1267
Anti-FLAG https://www.sigmaaldrich.cn/CN/zh/product/sigma/f1804
Goat anti-rabbit-HRP http://bioeasytech.com/product/2901.html?goods_id=5786
Goat anti-mouse-HRP http://bioeasytech.com/product/2907.html?goods_id=5794
Anti-BrdU antibody https://www.thermofisher.cn/cn/zh/antibody/product/BrdU-Antibody-clone-MoBU-1-Monoclonal/B35128
Anti-Rabbit-Alexa Fluor 555 https://www.abcam.cn/products/secondary-antibodies/goat-rabbit-igg-hl-alexa-fluor-555-ab150078.html
Goat-anti-mouse-DyLight 488 https://www.abcam.cn/products/secondary-antibodies/goat-mouse-igg-hl-dylight-488-ab96871.html

ChIP-seq

Data deposition

☒ Confirm that both raw and final processed data have been deposited in a public database such as GEO.

☒ Confirm that you have deposited or provided access to graph files (e.g. BED files) for the called peaks.

Data access links
May remain private before publication.

https://www.ncbi.nlm.nih.gov/geo/query/acc.cgi?acc=GSE216170

Files in database submission

Col-0_DMSO_rep1_R1.fastq.gz
Col-0_DMSO_rep2_R1.fastq.gz
Col-0_CPT_rep1_R1.fastq.gz
Col-0_CPT_rep2_R1.fastq.gz
atm_DMSO_rep1_R1.fastq.gz
atm_DMSO_rep2_R1.fastq.gz
atm_CPT_rep1_R1.fastq.gz
atm_CPT_rep2_R1.fastq.gz
asr20_DMSO_rep1_R1.fastq.gz
asr20_DMSO_rep2_R1.fastq.gz
asr20_CPT_rep1_R1.fastq.gz
asr20_CPT_rep2_R1.fastq.gz
pol2a_DMSO_rep1_R1.fastq.gz
pol2a_DMSO_rep2_R1.fastq.gz
pol2a_CPT_rep1_R1.fastq.gz
pol2a_CPT_rep2_R1.fastq.gz
Col-0_DMSO_rep1_R2.fastq.gz
Col-0_DMSO_rep2_R2.fastq.gz
Col-0_CPT_rep1_R2.fastq.gz
Col-0_CPT_rep2_R2.fastq.gz
atm_DMSO_rep1_R2.fastq.gz
atm_DMSO_rep2_R2.fastq.gz
atm_CPT_rep1_R2.fastq.gz
atm_CPT_rep2_R2.fastq.gz
asr20_DMSO_rep1_R2.fastq.gz
asr20_DMSO_rep2_R2.fastq.gz

asr20_CPT_rep1_R2.fastq.gz
asr20_CPT_rep2_R2.fastq.gz
pol2a_DMSO_rep1_R2.fastq.gz
pol2a_DMSO_rep2_R2.fastq.gz
pol2a_CPT_rep1_R2.fastq.gz
pol2a_CPT_rep2_R2.fastq.gz
Col-0_DMSO_rep1_fwd.bw
Col-0_DMSO_rep2_fwd.bw
Col-0_CPT_rep1_fwd.bw
Col-0_CPT_rep2_fwd.bw
atm_DMSO_rep1_fwd.bw
atm_DMSO_rep2_fwd.bw
atm_CPT_rep1_fwd.bw
atm_CPT_rep2_fwd.bw
asr20_DMSO_rep1_fwd.bw
asr20_DMSO_rep2_fwd.bw
asr20_CPT_rep1_fwd.bw
asr20_CPT_rep2_fwd.bw
pol2a_DMSO_rep1_fwd.bw
pol2a_DMSO_rep2_fwd.bw
pol2a_CPT_rep1_fwd.bw
pol2a_CPT_rep2_fwd.bw
Col-0_DMSO_rep1_rev.bw
Col-0_DMSO_rep2_rev.bw
Col-0_CPT_rep1_rev.bw
Col-0_CPT_rep2_rev.bw
atm_DMSO_rep1_rev.bw
atm_DMSO_rep2_rev.bw
atm_CPT_rep1_rev.bw
atm_CPT_rep2_rev.bw
asr20_DMSO_rep1_rev.bw
asr20_DMSO_rep2_rev.bw
asr20_CPT_rep1_rev.bw
asr20_CPT_rep2_rev.bw
pol2a_DMSO_rep1_rev.bw
pol2a_DMSO_rep2_rev.bw
pol2a_CPT_rep1_rev.bw
pol2a_CPT_rep2_rev.bw

Genome browser session
(e.g. UCSC)

https://genome.ucsc.edu/s/yujw/2022.11.09.liqin_bigwig

Methodology

Replicates

Two biologically independent replicates were used in seq assays, and the replicates highly correlated with each other as shown in the supplementary Figure.

Sequencing depth

Col-0_DMSO_rep1 ssDRIP-seq IP Col-0 DMSO_treatment paired-end,150bp 14577670
Col-0_DMSO_rep2 ssDRIP-seq IP Col-0 DMSO_treatment paired-end,150bp 19445813
Col-0_CPT_rep1 ssDRIP-seq IP Col-0 CPT_treatment paired-end,150bp 18096993
Col-0_CPT_rep2 ssDRIP-seq IP Col-0 CPT_treatment paired-end,150bp 14424553
atm_DMSO_rep1 ssDRIP-seq IP atm DMSO_treatment paired-end,150bp 16898302
atm_DMSO_rep2 ssDRIP-seq IP atm DMSO_treatment paired-end,150bp 18600208
atm_CPT_rep1 ssDRIP-seq IP atm CPT_treatment paired-end,150bp 19118697
atm_CPT_rep2 ssDRIP-seq IP atm CPT_treatment paired-end,150bp 16342977
asr20_DMSO_rep1 ssDRIP-seq IP asr20 DMSO_treatment paired-end,150bp 15158444
asr20_DMSO_rep2 ssDRIP-seq IP asr20 DMSO_treatment paired-end,150bp 16862235
asr20_CPT_rep1 ssDRIP-seq IP asr20 CPT_treatment paired-end,150bp 16184705
asr20_CPT_rep2 ssDRIP-seq IP asr20 CPT_treatment paired-end,150bp 14270715
pol2a_DMSO_rep1 ssDRIP-seq IP pol2a DMSO_treatment paired-end,150bp 16117456
pol2a_DMSO_rep2 ssDRIP-seq IP pol2a DMSO_treatment paired-end,150bp 13611015
pol2a_CPT_rep1 ssDRIP-seq IP pol2a CPT_treatment paired-end,150bp 14495061
pol2a_CPT_rep2 ssDRIP-seq IP pol2a CPT_treatment paired-end,150bp 15993988

Antibodies

S9.6 monoclonal antibody, purified from S9.6 hybridoma cell supernatant (ATCC HB-8730), was applied for ssDRIP-seq.

Peak calling parameters

The detailed ssDRIP-seq data analysis followed the pipeline in GitHub (https://github.com/PEHGP/ssDripPipeline). The main steps are described below: (1) fastqc was used to check the reads quality; (2) trim_galore was used to remove adapters and tails; (3) the reads were aligned to Tair10 with bowtie2; (4) Picard was used to remove duplicate reads; (5) samtools was used to split strands; (6) MACS2 was used to call R-loop peaks; (7) bamCoverage from deepTools was used to convert BAM format to bigwig format and normalized to 1 X sequencing depth.
The peak calling parameters were used as following: macs2 callpeak -t sample.bam -f BAMPE -g 119300826 -n sample.

Data quality

ssDRIP-seq peak signals of each dataset were visualized on genome browser. The narrow peak at <=5% FDR (default parameter) is from 12,000-19,000; and 100% of the peaks are above 5-fold enrichment compared to 1 X sequencing reads.

Software

FastQC v0.11.9 for quality control of reads
Trim_galore v0.6.5 for adapter cutting and tail trimming
Bowtie2 2.3.5.1 for alignment
bedtools v2.29.2 for genome annotation of ssDRIP peaks
Samtools 1.7 for strand splitting of ssDRIP-seq processing
Picard 2.24.2 for duplicates removing of ssDRIP reads
MACS2 2.2.7.1 for ssDRIP peak calling
DESeq2 1.32.0 for differentially expressed gene analysis of ssDRIP
deepTools 3.5.0 for nonquantified normalization and plotprofiling
